# Intensified Short Symptom Screening Program for Dengue Infection during Pregnancy, India

**DOI:** 10.3201/eid2604.191476

**Published:** 2020-04

**Authors:** Shilpa Naik, Matthew L. Robinson, Mallika Alexander, Ajay Chandanwale, Pradip Sambarey, Aarti Kinikar, Renu Bharadwaj, Gajanan N. Sapkal, Puja Chebrolu, Prasad Deshpande, Vandana Kulkarni, Smita Nimkar, Vidya Mave, Amita Gupta, Jyoti Mathad

**Affiliations:** Byramjee Jeejeebhoy Government Medical College and Sassoon General Hospitals, Pune, India (S. Naik, A. Chandanwale, P. Sambarey, A. Kinikar, R. Bharadwaj);; Johns Hopkins School of Medicine, Baltimore, Maryland, USA (M.L. Robinson, A. Gupta);; Byramjee Jeejeebhoy Government Medical College–Johns Hopkins Clinical Trials Unit, Pune (M. Alexander, P. Deshpande, V. Kulkarni, S. Nimkar, V. Maye);; National Institute of Virology, Pune (G.N. Sapkal);; Weill Cornell Medical College, New York, New York, USA (P. Chebrolu, J. Mathad)

**Keywords:** dengue, dengue virus, viruses, pregnancy, mosquito-borne diseases, screening program, infection, symptoms, vector-borne infections, India

## Abstract

Mosquitoborne diseases (e.g., malaria, dengue, and chikungunya) are endemic to India and pose diagnostic challenges during pregnancy. We evaluated an intensified short symptom screening program in India to diagnose dengue during pregnancy. During October 2017–January 2018, we screened pregnant women during antenatal surveillance for symptoms of mosquitoborne diseases (fever only, fever with conjunctivitis, fever with rash, or all 3 symptoms) within the previous 15 days. Of 5,843 pregnant women screened, 52 were enrolled and tested for dengue, chikungunya, and Zika viruses by using a Trioplex real-time reverse transcription PCR. Of 49 who had complete results, 7 (14%) were dengue positive. Of these ocular pain was seen in 4 (57%) and conjunctivitis in 7 (100%). Intensified symptom screening using conjunctivitis, in addition to rash, in pregnant women with fever might improve dengue case detection and can be included in routine symptom screening during pregnancy.

Every year, an estimated 96 million persons worldwide are given a clinical diagnosis of severe dengue infection (*1*). In 2017, a total of 188,401 cases of dengue were diagnosed in India, and the mortality rate was 0.1%. The mortality rate in Maharashtra State was 0.8% in 2017, nearly 8 times higher than the national average (*2**,**3*). The prevalence of dengue infection among pregnant women is not reported, but pregnant women may be uniquely susceptible to dengue infection because of the immune changes that occur during pregnancy (*4*).

In some countries in Southeast Asia, dengue fever is the most common cause of acute febrile illness during pregnancy (*5*). Dengue infection during pregnancy has been associated with poor maternal and infant outcomes, including preterm birth (*6*), hemorrhage, preeclampsia, and caesarean delivery (*7**–**15*). Dengue virus can also be vertically transmitted to the infant, resulting in neonatal dengue, thrombocytopenia, and cerebral hemorrhage (*16**–**19*). Vertical transmission is most likely if the infection occurs in the third trimester or is present during delivery by cesarean section (*20*).

Despite the devastating consequences, the diagnosis of dengue infection during pregnancy remains challenging. This challenge is partially caused by overlapping signs and symptoms of dengue infection with other conditions, such as HELLP (hemolysis, elevated liver enzymes, low platelet count) syndrome, pneumonia, pulmonary embolism, and other febrile illnesses (*21**,**22*). Moreover, molecular assays to diagnose dengue are not widely available. However, there is a remarkable reduction in the case-fatality rate after early diagnosis and access to appropriate medical care (*23*).

This study was planned after detection of Zika virus in India. The goal of this study was to determine the prevalence of dengue, chikungunya, and Zika virus among pregnant women in Pune, India. We also aimed to identify clinical predictors of these infections to improve screening and detection during the antenatal setting.

## Methods

### Ethics

This study was approved by the Byramjee Jeejeebhoy Medical College Clinical Trials Unit and Johns Hopkins University Institutional Review Boards. Written consent was obtained from all participants.

### Study Setting and Procedures

During October 2017–January 2018, we surveyed pregnant women who came to the antenatal clinic at Sassoon Government Hospital in Pune. All pregnant women were screened by a postgraduate physician who used a short symptom screening for fever, conjunctivitis, and rash occurring in the preceding 15 days (*24*). Women with any of these complaints underwent a secondary confirmatory screening by a senior physician. At this secondary screening, detailed histories, such as onset and duration of symptoms, reported by the women were collected. All women who had fever and conjunctivitis or fever and rash within the previous 15 days, confirmed in the secondary screening, were approached for enrollment in the study. After obtaining consent, research staff collected data on demographics, obstetric history, and travel history (*25*).

Blood samples were tested for dengue, chikungunya, and Zika viruses at the Indian National Institute of Virology by using Trioplex, a multiplex real-time reverse transcription PCR (RT-PCR) developed by the US Centers for Disease Control and Prevention (*26*). The primary physicians might have also ordered an IgM ELISA and a nonstructural protein 1 antigen test to assess for dengue or chikungunya infection if there was clinical suspicion for either infection. We abstracted this information from medical charts.

We completed follow-up visits at delivery and 6 months postpartum. For women who delivered outside Sassoon Hospital, we abstracted data from their medical records through home visits or postpartum follow-ups. Infants born to enrolled mothers had follow-up visits at birth and 6 months of age to coincide with maternal visits. We planned for additional evaluations if any abnormality was detected.

All data collected were stored on a secure electronic database. This database was specifically designed for our study by Persistent Systems (https://www.persistent.com) on a Salesforce platform (https://www.salesforce.com) by using tablets or laptops.

### Sample Size

We did not have a predetermined sample size, but screened all women who came to the center during October 2017–January 2018. A total of 5,843 patients were screened for enrollment.

## Results

Among 5,843 pregnant women who attended antenatal visits during the study period, 106 (2%) reported fever, rash, or conjunctivitis in the 15 days preceding their visit in the primary screening. Of these 106 women, we enrolled 52 (49%) who were found to be eligible in the secondary screening by the medical team. These women had the following symptoms: 18 (34%) fever only, 4 (7%) fever plus rash, 18 (34%) fever plus conjunctivitis, and 12 (23%) all 3 symptoms (Figure). We screened out the remaining 54 patients during the secondary screening because they had their symptoms >15 days before the visit, which were our inclusion criteria.

**Figure:**
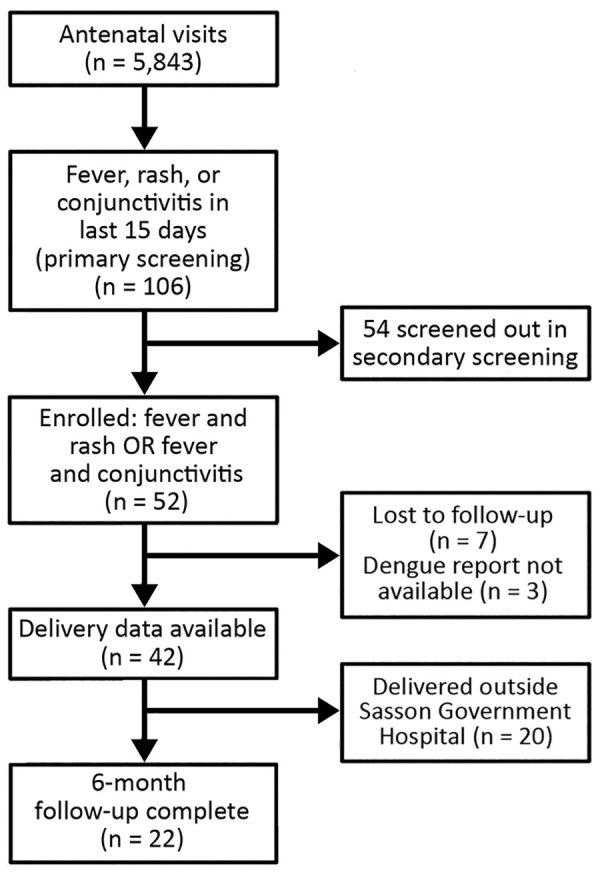
Screening and enrollment flowchart for participation in an intensified short symptom screening program for dengue infection during pregnancy, India.

The median age of enrolled participants was 22 years (interquartile range [IQR] 19–25 years). The median gestational age at enrollment was 23 weeks (IQR 18–34 weeks); half of the participants were pregnant for the first time. The most common symptoms reported by participants were headache (n = 33/52, 63%), cough (n = 26/52, 50%), conjunctivitis (n = 26/52, 50%), and rash (n = 13/52, 25%) (Table 1).

**Table 1:** Clinical characteristics for 52 pregnant women with fever during intensified short symptom screening program for dengue infection during pregnancy, India*

Clinical characteristic	Entire population, n = 52	Maternal dengue PCR positive, n = 7	Maternal dengue PCR negative, n = 42†	OR (95% CI)	p value
Age	22 (19–25)	20 (19–23.5)	22.5 (19.2–25)	0.9 (0.7–1.0)	0.42
Gestational age at enrollment	23 (18–34)	30 (22.5–31.5)	23 (16.5–34)	0.1 (0–0.9)	0.57
Primigravida	25 (48)	4 (57)	21 (50)	1.3 (0.2–10.2)	1
HIV positive	2 (4)	0 (0)	2 (5)‡	0 (0–33.9)	1
Recent travel	15 (29)	1 (14)	14 (33)	0.3 (0–3.2)	0.41
Signs/symptoms					
Myalgia	18 (35)	3 (43)	15 (36)	1.3 (0.2–9.1)	0.7
Body pain	5 (10)	1 (14)	4 (10)	1.6 (0–20.1)	0.55
Arthralgia	13 (25)	3 (43)	10 (24)	2.4 (0.3–16.7)	0.36
Weakness	3 (6)	1 (17§	2 (5)¶	3.5 (0.1–80.7)	0.36
Malaise	2 (4)	1 (14)	1 (2)§	6.2 (0.1–533.1)	0.27
Lymphadenopathy	0 (0)	0 (0)	0 (0)§	NA	NA
Abdominal pain	1 (2)	1 (14)	0 (0)§	∞ (0.2–∞)	0.15
Vomiting	14 (27)	3 (43)	11 (26)	2.1 (0.3–14.6)	0.39
Diarrhea	5 (10)	1 (14)	4 (10)§	1.5 (0–19.6)	0.56
Poor appetite	14 (27)	3 (43)	11 (27)§	2 (0.3–14.1)	0.4
Sore throat	16 (31)	1 (14)	15 (36)	0.3 (0–2.9)	0.4
Eye pain	10 (19)	4 (57)	6 (14)	7.5 (1–65.6)	0.02
Conjunctivitis	26 (50)	7 (100)	19 (46)§	∞ (1.4–∞)	0.01
Rash	13 (25)	2 (29)	11 (26)	1.1 (0.1–8.2)	1
Headache	33 (63)	6 (86)	27 (64)	3.3 (0.3–163.2)	0.4
Cough	26 (50)	3 (43)	23 (56)§	0.6 (0.1–4)	0.69

*Values are no. (%) or no. (range). NA, not applicable; OR, odds ratio. †Trioplex results were not available for 3 women. ‡Incomplete symptom data for 5 participants. §Incomplete symptom data for 1 participant. ¶Incomplete symptom data for 3 participants.

Trioplex testing reports were available for 49/52 (94%) participants. Positive results for dengue were obtained for 7/49 (14%) participants. Per routine clinical care, a few patients underwent immunoassay testing for dengue (n = 4) and chikungunya (n = 3). Of the 7 women who were positive for dengue by Trioplex, 1 (14%) showed negative results by a rapid diagnostic test and 1 (14%) showed positive results by IgM ELISA. Among the remaining 42 women who were negative for dengue by Trioplex, 1 (2%) was negative by a dengue rapid diagnostic test and 1 (2%) was positive by IgM ELISA. All samples showed negative results for Zika and chikungunya viruses by Trioplex. A chikungunya IgM ELISA was performed for 3 participants; 2 (4%) had positive results (Table 2).

**Table 2:** Trioplex and standard of care test results during intensified short symptom screening program for dengue infection during pregnancy, India

Trioplex results	Standard of care results
Dengue positive, n = 7	1 rapid diagnostic negative, 1 IgM positive, 5 not tested
Dengue negative, n = 42	1 rapid diagnostic negative, 1 IgM positive, 40 not tested

We found no major difference in demographic features between participants who were positive for dengue and those who were negative (Table 1). Symptomatically, dengue diagnosis was associated with eye pain (OR 7.5, 95% CI 1–65.6; p = 0.02). All women given a diagnosis of dengue had conjunctivitis (p = 0.01), and 2 women (29%) had rash.

Data collected at the time of delivery were available for 42 (85%) patients; we were unable to obtain delivery date for 7 (15%) patients. The median gestational age at the time of delivery was 38 weeks (IQR 37–39 weeks), which did not differ between mothers who had been given a diagnosis of dengue and those who had not (Table 2). Ten (24%) births were performed by cesarean section. There were 9 (21%) premature infants, 10 (24%) low birth weight infants, 2 (5%) pregnancies complicated by oligohydramnios, and 9 (21%) infants with head circumferences less than the third percentile; none of these findings were associated with maternal dengue infection. There was 1 (2%) stillbirth to a dengue-negative mother. By the time of 6 months follow-up, 3 infant deaths were recorded. Maternal dengue infection was not associated with any of the observed adverse birth outcomes (Table 3).

**Table 3:** Birth outcomes by maternal dengue infection status among 42 deliveries analyzed by intensified short symptom screening program for dengue infection during pregnancy, India*

Clinical characteristic	All births, n = 42	Maternal dengue positive, n = 6	Maternal dengue negative, n = 34	OR (95% CI)	p value
Gestational age, wk	38 (37–39)	37.7 (37.2–38.1)	39.1 (37.9–39.9)	1.0 (0.7–1.5)	0.28
Cesarean section	10 (24)	1 (17)	9 (26)	0.6 (0–6.1)	1
Premature	9 (21)	1 (17)	8 (24)	0.7 (0–7.3)	1
Low birthweight	10 (24)	2 (33)	7 (21)	1.9 (0.1–16.8)	0.60
Head circumference <3rd percentile†	9 (21)	2 (33)	7 (22)‡	1.8 (0.1–15.6)	0.60
Head circumference <10th percentile†	14 (33)	2 (33)	12 (38)‡	0.8 (0.1–6.9)	1
Stillbirth	1 (2)	0 (0)	1 (3)	0 (0–220.2)	1
Deceased at birth or before 6-mo follow-up§	3 (7)	0 (0)	3 (9)	0 (0–15)	1
Oligohydramnios	2 (5)	1 (17)	1 (3)	6.1 (0.1–530.4)	0.28
Apgar score at 1 min	8 (7–8)	8 (8–8)	8 (7–8)	2.1 (0.4–16.2)	0.36
Apgar score at 5 min	9 (9–9)	9 (9–9)	9 (9–9)	0.9 (0.3–6.7)	0.65

*Values are no. (%) or no. (range). OR, odds ratio.  †Intergrowth standards.  ‡Head circumference data were not available for 2 infants. §Six-month follow-up data were available for 22 infants.

## Discussion

We report a dengue prevalence of 14% among pregnant women with rash or conjunctivitis and a history of recent fevers. However, we did not identify chikungunya and Zika cases in our study. The prevalence of dengue infection in pregnant women, in general, is not reported in the literature, but the prevalence of laboratory-confirmed dengue among persons with clinically suspected cases in the population in India was reported to be 38% (*27*).

The lower prevalence in our population could reflect our short enrollment period, which did not include the full peak monsoon season because the reports of Zika in India only began in May 2017. Another explanation might be related to the diagnostic method we used. The RT-PCR used in this study identifies dengue infection in the acute phase of infection within 5 days of infection, which coincides with viremia and the febrile phase of illness (*28*). Although RT-PCR is the most sensitive test for diagnosis of acute dengue and can distinguish between dengue and Zika, its high cost and complexity precludes its use for routine care in India. Most studies in India reported diagnosis of dengue by using serologic tests (IgM and IgG), which are less sensitive in early infection but become more sensitive after 5 days of infection (*29*). Regardless, a prevalence rate of 14% is a cause for concern, given possible adverse maternal–fetal outcomes.

A useful finding of our study was the predictive value of ocular findings in the diagnosis of dengue in our pregnant cohort. A separate study of pregnant women with dengue in northern India reported similar findings: 100% of the dengue patients had conjunctivitis, and 57% had eye pain (*30*). According to the World Health Organization, dengue should be considered in patients with a high fever and retroorbital pain (*31**–**34*), but there is no specific mention of conjunctivitis as a trigger for dengue testing. The mechanism behind dengue infection and ophthalmic involvement is not clear, but studies suggest it is related to an immune-mediated process involving specific dengue serotypes (*31**,**32*). Given the results of our studies and others for pregnant women, conjunctivitis should be added to screening for dengue in pregnancy during high-burden months.

In our study, we found that 29% of women with dengue had preterm births and low birthweight infants, which is consistent with recent reviews reporting these factors as the 2 most common adverse pregnancy outcomes (*9*). Although 9 (21%) infants had head circumferences less than the third percentile, this finding was not associated with maternal dengue infection. We also did not find any adverse outcomes related to bleeding in the mother or the neonate; thrombocytopenia was not seen in our group of patients. This finding is different from those of most studies in which postpartum hemorrhage and disseminated intravascular coagulation have been reported. Other obstetric complications, including preeclampsia, eclampsia, placenta previa, or retroplacental hematoma, have been reported in the literature. The gestational age at manifestation of dengue fever results in a major effect on outcomes; manifestations with early or late onset during pregnancy are associated with the worst prognosis (*35*).

Immunologic changes that occur in mid-to-late pregnancy could lead to an increase in the risk for severe dengue if infections occur during pregnancy (*9*,*36*,*37*). In our study, women were screened mainly during the second and third trimesters because the woman usually come for antenatal care during that period. Moreover, none of the patients in this study had placenta previa, abruptio placentae, or severe preeclampsia associated with thrombocytopenia, thus avoiding bleeding and need for transfusions, although preeclampsia was present in 2 of the dengue patients. The 1 stillbirth that occurred was the result of the umbilical cord being wrapped around the neck of the infant and is unlikely to have been related to maternal dengue.

Other studies have also reported a later diagnosis of dengue during pregnancy; a study in Sri Lanka reported 96% of cases in the second or third trimester (*38*). The reason why illness onset usually occurs in mid-to-late pregnancy is unclear. One reason that dengue is not diagnosed easily in early pregnancy is because women do not come to a clinic early or they believe that their symptoms are normal for early pregnancy (e.g., fever, nausea, vomiting). Some patients might not have been aware of their pregnancies in the first several weeks after conception and therefore are less likely to recall a febrile episode than a patient with a known pregnancy. The physiologic and immunologic changes that occur in mid-to-late pregnancy might also contribute to an increase in dengue virus susceptibility. Because dengue infection is common in India, active symptom-based screening of all pregnant women should be incorporated into antenatal care during the high-risk months. Our implementation of an active screening algorithm may have decreased the incidence of adverse pregnancy and birth outcomes.

Our study had several limitations. First, the number of cases detected was small, which might limit detection of major associations with maternal and neonatal outcomes. Because we conducted the study only during September–February, the full temporal pattern of dengue and its complications in pregnancy might have been missed. For this study, because we did not enroll afebrile women with ophthalmic symptoms, we were not able to assess if these symptoms are independently predictive of dengue infection in pregnancy. We plan to incorporate dengue screening into antenatal care in the coming years and with longer infant follow-up to improve the yield of this intervention. However, even with these limitations, we were still able to identify 7 cases of dengue, suggesting that scaling up the study would result in an even higher detection rate.

Although the primary purpose of conducting this study was Zika surveillance, we instead identified 7 cases of dengue on the basis of similar symptoms. This finding suggests that intensified symptom screening by using conjunctivitis, in addition to rash, in pregnant women with fever might increase the efficiency of dengue case detection. The symptom screening is short and can be administered by ancillary medical staff with minimal training to detect cases in the field or in a busy antenatal clinic. Given that the mortality rate for severe dengue fever is 0.8%–2.5% (*2*), this type of active symptom-based screening for dengue might help prevent adverse maternal and fetal outcomes.
